# A retrospective population-based study of induction of labour trends and associated factors among aboriginal and non-aboriginal mothers in the northern territory between 2001 and 2012

**DOI:** 10.1186/s12884-016-0899-7

**Published:** 2016-05-31

**Authors:** Pasqualina Coffey, John Condon, Karen Dempsey, Steven Guthridge, Fintan Thompson

**Affiliations:** Health Gains Planning Branch, Department of Health, Darwin, Australia; Menzies School of Health Research, Charles Darwin University, Darwin, Australia; Centre for Chronic Disease Prevention, The Cairns Institute, James Cook University, Cairns, Australia

**Keywords:** Induction of labour, Pregnancy, Indigenous

## Abstract

**Background:**

Induction of labour (IOL) has become more common among many populations, but the trends and drivers of IOL in the Northern Territory (NT) of Australia are not known. This study investigated trends in IOL and associated factors among NT Aboriginal and non-Aboriginal mothers between 2001 and 2012.

**Methods:**

A retrospective analysis of all NT resident women who birthed in the NT between 2001 and 2012 at ≥32 weeks gestation. Demographic, medical and obstetric data were obtained from the NT Midwives’ Collection. The prevalence of IOL was calculated by Aboriginal status and parity of the mother and year of birth. The prevalence of each main indication for induction among women was compared for 2001–2003 and 2010–2012. Linear and logistic regression was used to test for association between predictive factors and IOL in bivariate and multivariate analysis, separately for Aboriginal and non-Aboriginal mothers.

**Results:**

A total of 42,765 eligible births between 2001 and 2012 were included. IOL was less common for Aboriginal than non-Aboriginal mothers in 2001 (18.0 % and 25.1 %, respectively), but increased to be similar to non-Aboriginal mothers in 2012 (22.6 % and 24.8 %, respectively). Aboriginal primiparous mothers demonstrated the greatest increase in IOL. The most common indication for IOL for both groups was post-dates, which changed little over time. Medical and obstetric complications were more common for Aboriginal mothers except late-term pregnancy. Prevalence of diabetes in pregnancy increased considerably among both Aboriginal and non-Aboriginal mothers, but was responsible for only a small proportion of IOLs. Increasing prevalence of risk factors did not explain the increased IOL prevalence for Aboriginal mothers.

**Conclusions:**

IOL is now as common for Aboriginal as non-Aboriginal mothers, though their demographic, medical and obstetric profiles are markedly different. Medical indications did not explain the recent increase in IOL among Aboriginal mothers; changes in maternal or clinical decision-making may have been involved.

## Background

Induction of labour (IOL) is the artificial initiation of labour and is undertaken when continuing a pregnancy is associated with a greater level of maternal or fetal risk [1]. IOL has become more common in many populations around the world [1], which has been well documented in the United States of America [2, 3], Europe [4, 5], the United Kingdom [6, 7] and some states of Australia [8–10]. While there are advantages to inducing labour under certain circumstances such as pregnancies over 41 weeks gestation [11] or maternal hypertension [12], there is contention surrounding the wider practice. In some instances, increasing rates of IOL have not been adequately explained by evidence-based indications [13–15] nor associated with improved maternal or neonatal outcomes [15, 16]. There are also differing findings regarding the likelihood of caesarean section following induction of labour compared to expectant management [17, 18], with high caesarean section rates observed in primiparous mothers undergoing IOL [16].

The Northern Territory (NT) has a different population profile from the rest of Australia. Aboriginal people make up almost 30 % of the NT’s population [19], the highest of any jurisdiction in Australia. The NT also has a younger and more fertile population compared with Australia as a whole [20]. Complications during pregnancy and adverse birth outcomes are more common among Aboriginal than non-Aboriginal women, including: medical conditions such as diabetes and hypertension; health risk behaviours such as smoking and late presentation for antenatal care; pregnancy complications such as intrauterine growth restriction; and fetal death [21]. Few studies have investigated trends and drivers of obstetric intervention specifically among Aboriginal mothers in Australia. Generally these have demonstrated lower intervention rates among Aboriginal mothers compared to non-Aboriginal mothers, and poorer maternal and infant health outcomes [22, 23]. There have been no focussed studies of IOL among NT mothers.

It is important to explore the trends in IOL to better understand the drivers and associated factors in a variety of settings. This study aims to compare the trends in IOL among NT Aboriginal and non-Aboriginal mothers between 2001 and 2012 and to identify the socio-demographic, medical and obstetric factors associated with these trends.

## Methods

This was a retrospective population-based analysis of all NT resident women who birthed in the NT between 2001 and 2012 and laboured at ≥32 weeks gestation. In this study, the term ‘Aboriginal’ is used to refer to people of Aboriginal and Torres Strait Islander origin.

Data were obtained from the NT Midwives’ Collection (MC), a statutory population-based census of all births in the NT of at least 20 weeks gestation or with a birth weight of at least 400 g. The MC contains information on maternal and neonatal characteristics, and important factors affecting the pregnancy, labour process and delivery outcomes. Some demographic data, including Aboriginal status is taken directly from the hospital patient information software (CareSys). Midwives in public hospitals enter information shortly after the birth of a baby via the Birthing Suite Module of the hospital information system. Births from the NT’s only private hospital and planned home births are entered via the Internet. Information regarding other out of hospital births are submitted in paper form and entered by the MC Perinatal Business Analyst. Data were extracted from the MC using SAP Business Objects (SAP, USA). Data analysis was performed using Stata version 13.0 (Statacorp, College Station, Texas, USA).

Type of labour was defined as “induced” if this was recorded as the onset of delivery, or if a main indicator for induction and induction method were both listed in the record. The MC allows entry of one ‘main indication’ of induction from a list of 12 options.

Mothers were classified as urban if they resided in one of the five regional cities/towns in the NT: Darwin/Palmerston and its hinterland, Alice Springs, Katherine, Nhulunbuy and Tennant Creek. These are the towns in the NT that have a hospital with maternity services. Place of birth in a public hospital included births at all the public hospitals located in these towns. Private included births from the Darwin Private Hospital. ‘Other’ place of birth included births in community health centres, home births, and births in transit. Women were classified as having smoked during pregnancy if smoking was recorded at any time during their pregnancy. Early antenatal visit was defined as having the first antenatal visit before 14 weeks gestation. For marital status, the other classification included women who were divorced, widowed, or had ‘other’ status in the MC.

Mothers were classified as having hypertension if this was recorded as a pre-existing medical condition, a complication of labour, or as an indicator for induction. Pre-eclampsia was classified separately (i.e. mothers with a recorded diagnosis of pre-eclampsia were not included in the hypertension group). Diabetes in pregnancy included both pre-existing and gestational diabetes. Macrosomia was classified as birth weight ≥ 4000 g. Intrauterine growth restriction was based on birth weight below the 10th percentile for gestational age and gender. Preterm delivery was defined as delivery before 37 completed weeks gestation. The definition of ‘post-term’ in the MC was at or beyond 42 weeks gestation, however initial data validation showed that inductions for post-term pregnancies were most frequently undertaken at 41 weeks, so late-term was defined as pregnancy at or greater than 41 completed weeks gestation.

Of the 44, 899 NT women who birthed between 2001 and 2012, 913 (2.0 %) were before 32 weeks gestation and 1117 (2.5 %) were interstate mothers and were excluded. Women were also excluded if they were missing key data. This resulted in 104 (0.2 %) exclusions for missing: Aboriginal status (*n =* 1); birth weight (*n =* 2); gestation (*n =* 6); maternal age (*n =* 1); parity (*n =* 8); place of birth (*n =* 1); presentation of the fetus (*n =* 68); and residence (*n =* 17).

### Statistical analysis

The prevalence of IOL was calculated as the number of induced labours divided by the total number of women who birthed (greater than or equal to 32 weeks gestation). The proportional change in IOL was calculated as the difference in the proportion of IOLs in 2001–2003 and 2010–2012 divided by the proportion in 2001–2003.

The prevalence of each main indication for induction among women who were induced was compared (separately for Aboriginal and non-Aboriginal mothers) for the first three years (2001–2003) and last three-years (2010–2012) of the study period.

Bivariate analyses of the association between predictive factors and IOL were performed separately for Aboriginal and non-Aboriginal women because the prevalence of some predictive factors and the association between some of these factors and IOL was found to be different for Aboriginal than non-Aboriginal women. Generalised linear regression was used to calculate odds ratios (OR) to test for association.

Multivariable logistic regression analysis was used to assess association between prevalence of IOL and multiple predictive factors, separately for Aboriginal and non-Aboriginal mothers. All variables were included in an initial logistic regression. If the p-value of the OR was >0.05, or if the p-value was <0.05 but the OR was close to 1.0 (between 0.90 and 1.10) and authors felt that the variable was not of clinical significance, the variable was not included in the final regression model. The same model was used for Aboriginal and non-Aboriginal mothers and included the following variables: first-time mother; ≥ 3 previous births; private hospital; early antenatal visit; previous caesarean section; diabetes in pregnancy; hypertension; pre-eclampsia; premature rupture of membranes; prolonged rupture of membranes; late term; malpresentation; macrosomia; and year.

The study was approved by the Human Research Ethics Committee of the Northern Territory Department of Health and the Menzies School of Health Research (HREC reference 2013–2087).

## Results

A total of 42,765 births between 2001 and 2012 were included in the analysis, comprising 15,730 Aboriginal and 27,035 non-Aboriginal mothers (Table 1).

**Table 1:** Social, medical and obstetric factors of Northern Territory women who birthed in 2001–2012 by Aboriginal status and induction of labour status

	Aboriginal mothers			Non-Aboriginal mothers		
	Induced	Not induced	OR (95 % CI)	Induced	Not induced	OR (95 % CI)
Total births (number, %)	3012 (19.1)	12718 (80.9)		6570 (24.3)	20465 (75.7)	
Characteristics of mothers	%	%		%	%	
Maternal age						
Less than 20 years	25.6	25.1	1.05 (0.93, 1.18)	4.0	4.4	0.88 (0.76, 1.01)
20 to 24 years	28.3	32.9	0.89 (0.79, 0.99)	16.6	16.0	1.00 (0.92, 1.09)
25 to 29 years	21.9	22.6	1.00	29.8	28.8	1.00
30 to 34 years	15.5	12.9	1.25 (1.09, 1.42)	30.4	31.1	0.95 (0.88, 1.02)
35 years and over	8.7	6.5	1.37 (1.17, 1.62)	19.1	19.7	0.94 (0.86, 1.02)
Marriage status						
Single	49.7	54.5	0.83 (0.76, 0.89)	17.3	17.8	1.02 (0.94, 1.09)
Married/defacto	45.2	41.0	1.00	63.8	66.6	1.00
Other	5.2	4.6	1.03 (0.85, 1.24)	18.9	15.6	1.26 (1.17, 1.36)
Parity						
First-time mother	40.4	28.9	1.86 (1.70, 2.05)	51.4	41.3	1.55 (1.46, 1.65)
1-2 previous births	34.3	45.6	1.00	39.8	49.7	1.00
≥3 previous births	25.3	25.5	1.32 (1.19, 1.47)	8.7	8.9	1.22 (0.25, 0.27)
Place of birth						
Public hospital	98.9	94.4	1.00	62.5	70.9	1.00
Private hospital	1.1	0.6	1.71 (1.21, 2.70)	37.5	26.9	1.58 (1.49, 1.68)
Other	0.0	5.0	NA^a^	0.0	2.2	0.02 (0.00, 0.06)
Residence of mother						
Urban	30.0	28.7	1.00	88.9	87.8	1.00
Remote	70.0	71.3	0.94 (0.86, 1.03)	11.1	12.2	0.90 (0.82, 0.98)
Medical and obstetric factors^b^						
Multiple pregnancy	0.8	0.9	0.88 (0.56, 1.38)	1.0	1.3	0.72 (0.55, 0.95)
Early antenatal visit	47.8	43.3	1.20 (1.11, 1.30)	77.1	74.1	1.18 (1.10, 1.26)
Previous caesarean section	7.4	20.4	0.31 (0.27, 0.36)	3.6	17.9	0.17 (0.15, 0.19)
Smoked during pregnancy	38.1	44.8	0.76 (0.70, 0.82)	15.0	16.7	0.88 (0.81, 0.95)
Diabetes in pregnancy	17.4	8.0	2.42 (2.16, 2.71)	7.6	5.2	1.51 (1.35, 1.68)
Maternal hypertension	7.5	2.5	3.13 (2.62, 3.73)	4.5	1.2	4.00 (3.36, 4.76)
Pre-eclampsia	14.4	3.1	5.27 (4.56, 6.07)	8.5	2.0	4.59 (4.02, 5.23)
Antepartum haemorrhage	1.6	1.7	0.93 (0.68, 1.27)	2.1	2.4	0.88 (0.73, 1.07)
Malpresentation	1.0	4.9	0.19 (0.13, 0.28)	1.1	6.0	0.18 (0.14, 0.23)
Preterm delivery	10.4	12.3	0.83 (0.73, 0.94)	3.8	6.3	0.58 (0.51, 0.67)
Late term (≥41 weeks)	24.4	5.9	5.18 (4.64, 5.79)	27.9	8.5	4.15 (3.86, 4.46)
Premature rupture of membranes	5.8	2.8	2.10 (1.74, 2.53)	2.2	1.4	1.63 (1.33, 2.00)
Prolonged rupture of membranes	16.5	3.1	6.20 (5.39, 7.12)	9.3	1.7	6.04 (5.27, 6.92)
Intrauterine growth retardation	18.5	17.7	1.05 (0.95, 1.17)	9.3	9.3	1.00 (0.91, 1.10)
Macrosomia	10.8	6.7	1.69 (1.47, 1.93)	16.0	10.9	1.55 (1.43, 1.68)

^a^Odds ratio not applicable as there were no cases among induced mothers

^b^Medical and obstetric factors compared mothers with the factor to those without the factor

**Fig. 1:**
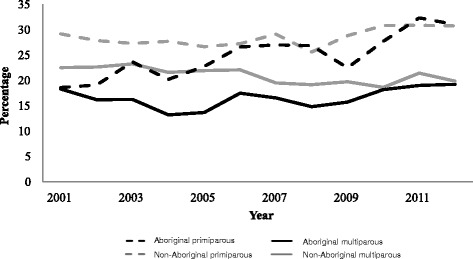
Trends in induction of labour 2001–2012 by status and parity

IOL was more common for primiparous than multiparous mothers among both Aboriginal and non-Aboriginal mothers (Fig. 1, Table 2). For non-Aboriginal mothers, the prevalence of IOL increased to a small extent for primiparous mothers but decreased by a similar extent for multiparous mothers, resulting in little overall change. IOL prevalence was higher among non-Aboriginal than Aboriginal mothers in 2001, but by 2012 the rates had converged for both primiparous and multiparous mothers, with the greatest increase seen among Aboriginal primiparous mothers.

**Table 2:** Proportion of women who had an induction of labour by Aboriginal status and parity, 2001–2003 and 2010-2012

	2001–2003		2010–2012		Change in proportion between 2001–2003 and 2010–2012 %
	Number	%	Number	%	
Aboriginal	712	18.0	881	22.6	25.1
Primiparous	256	20.4	386	30.3	48.9
Multiparous	456	16.9	495	18.8	11.0
Non-Aboriginal	1639	25.1	1801	24.8	−1.4
Primiparous	799	28.1	990	30.8	9.5
Multiparous	840	22.8	811	20.0	−12.4

Bivariate analysis for both Aboriginal and non-Aboriginal women demonstrated that IOL was more common for mothers who: birthed in a private hospital; were primiparous or had three or more previous births; had early first antenatal visit; had diabetes, hypertension or pre-eclampsia during pregnancy; had a pregnancy of 41 or more weeks gestation; had premature or prolonged rupture of membranes; or had macrosomia (Table 1). IOL was less common for mothers with: previous caesarean section(s); multiple pregnancies; smoking during pregnancy; malpresentation; or a pre-term delivery. For Aboriginal mothers only, the rate of IOL was higher among mothers aged 30 and over, and lowest among those aged 20 to 24. IOL was also less common for Aboriginal single mothers. IOL was less common among those living in a remote area, but this was only significant for non-Aboriginal mothers.

The proportion of births that were vaginal deliveries decreased between 2001–2003 and 2010–2012 while the proportions of emergency and elective caesarean sections increased, for both Aboriginal and non-Aboriginal mothers (Table 3). Demographic changes between 2001 and 2012 included an increase in the proportion of older mothers, and a decrease in the proportion of mothers who were married or urban residents. The proportion of non-Aboriginal women whose marital status was ‘other’ jumped significantly between the first and last periods of the study, which may represent an anomaly in data reporting. Smoking prevalence fell by almost half for non-Aboriginal mothers but did not decrease for Aboriginal mothers. By 2010–2012 smoking was four times more common among Aboriginal than non-Aboriginal mothers.

**Table 3:** Characteristics of all Northern Territory residents who birthed: comparison of time periods 2001–2003 and 2010–2012, by Aboriginal status

	Aboriginal		Non-Aboriginal	
	2001-2003	2010-2012	2001-2003	2010-2012
Total births (n)	3,949	3,905	6,524	7,271
	% of births	% of births	% of births	% of births
Induction of labour	18.0	22.6	25.1	24.8
Vaginal delivery (including instrumental)	76.3	72.0	73.7	68.5
Elective caesarean	7.5	10.0	12.9	16.2
Emergency caesarean	16.2	18.0	13.4	15.4
Characteristics of mothers				
Maternal age				
Less than 20 years	29.7	22.1	5.5	3.1
20 to 24 years	30.9	32.2	17.2	15.3
25 to 29 years	21.6	23.5	29.5	29.8
30 to 34 years	12.3	14.3	31.3	31.0
35 years and over	5.5	8.0	16.6	20.8
Marriage status				
Single	48.6	60.6	19.4	17.8
Married/defacto	44.6	36.4	74.7	60.3
Other	6.8	3.0	5.9	21.8
Parity				
First-time mother	31.8	32.6	43.5	44.2
1–2 previous births	42.9	42.6	47.1	47.8
≥ 3 previous births	25.2	24.8	9.3	8.0
Place of birth				
Public hospital	94.5	95.6	68.9	70.2
Private hospital	1.2	0.4	29.7	27.9
Other	4.2	3.9	1.3	1.9
Residence of mother				
Urban	31.2	27.3	92.9	85.7
Remote	68.8	72.7	7.1	14.3
Medical and obstetric factors				
Multiple pregnancy	0.8	1.0	1.3	1.2
Early antenatal visit	38.4	50.3	64.4	82.0
Previous caesarean section	17.0	18.4	12.0	15.8
Smoked during pregnancy	40.5	46.5	20.6	11.1
Diabetes in pregnancy	6.2	13.2	3.3	7.9
Maternal hypertension	4.3	3.3	2.1	1.6
Pre-eclampsia	5.2	5.3	4.0	2.3
Antepartum haemorrhage	1.7	1.6	2.7	2.4
Malpresentation	5.1	4.1	4.9	4.0
Preterm delivery	12.2	12.0	6.0	5.3
Late-term (≥41 weeks)	8.8	9.1	13.2	14.7
Premature rupture of membranes	4.6	3.2	1.5	1.6
Prolonged rupture of membranes	6.0	5.6	3.5	3.8
Intrauterine growth retardation	18.2	17.0	10.0	8.6
Macrosomia	6.4	7.8	11.3	13.1

The prevalence of maternal diabetes more than doubled between 2001–2003 and 2010–2012 for both Aboriginal and non-Aboriginal women (Table 3). Late-term pregnancies and macrosomia also increased for both groups, while pre-eclampsia increased marginally among Aboriginal mothers only. All other medical and obstetric complications decreased for both Aboriginal and non-Aboriginal mothers.

‘Post-dates’ was the most common reason for IOL recorded in the MC for both groups between 2001 and 2012, followed by ‘hypertension’ for Aboriginal mothers and ‘other’ for non-Aboriginal mothers (Table 4). IOL for post-dates was carried out most commonly at 41 weeks (66.9 % and 62.4 % of these IOLs for Aboriginal and non-Aboriginal mothers respectively)(data not shown). The greatest increase among the IOL indications for both Aboriginal and non-Aboriginal mothers was ‘diabetes’, while ‘unknown’ showed the greatest decrease. Indications that became less common were ‘social reasons’ and ‘hypertension’ for non-Aboriginal mothers and ‘prolonged rupture of membranes’ and ‘intrauterine growth restriction’ for Aboriginal mothers (Table 4).

**Table 4:** Main reason for induction of labour among Northern Territory Aboriginal and non-Aboriginal mothers, 2001–2012

	Aboriginal mothers				Non-Aboriginal mothers			
Main indication	2001–2003 (%)	2010–2012 (%)	Overall %	Average annual % change in OR (95 % CI)	2001–2003 (%)	2010–2012 (%)	Overall %	Average annual % change in OR (95 % CI)
Hypertension	17.6	18.6	17.5	0.3 (–2.4, 3.0)	12.3	6.6	10.1	–6.2 (–8.3,–4.0)
IUGR	9.8	7.5	8.5	–3.7 (–7.2,–0.2)	3.0	2.7	3.3	–2.4 (–6.1, 1.4)
Post-dates	24.3	24.5	27.6	–0.2 (–2.4, 2.1)	33.4	37.3	35.5	2.7 (1.3, 4.2)
Diabetes	6.2	11.9	9.3	9.2 (5.3, 13.2)	2.5	7.1	4.7	11.3 (7.6, 15.2)
Premature ROM	3.7	3.9	3.4	0.4 (–5.1, 6.3)	1.3	2.2	1.5	7.3 (1.3, 13.6)
Prolonged ROM	16.4	10.9	12.9	–4.4 (–7.2,–1.5)	8.3	8.6	7.8	–0.4 (–3.0, 2.1)
Fetal death in utero	0.6	1.1	1.0	3.7 (–6.3, 14.8)	0.5	0.2	0.5	–7.1 (–15.5, 2.3)
Social reason	2.5	2.2	2.4	–0.1 (–6.6, 6.8)	11.7	6.4	9.3	–6.8 (–9.0,–4.5)
Other	18.0	19.5	17.1	0.9 (–1.8, 3.7)	22.5	28.8	25.1	3.3 (1.7, 5.0)
Unknown	1.0	0.1	0.4	–22.2 (–35.7,–5.8)	4.5	0.1	2.1	–23.3 (–27.7,–18.6)

IUGR intra uterine growth restriction; ROM Rupture of membranes

After adjustment, several factors remained strongly associated with increased prevalence of IOL for both Aboriginal and non-Aboriginal mothers: prolonged rupture of membranes, pre-eclampsia, late-term pregnancy, diabetes, hypertension, pre-labour rupture of membranes and a private hospital birth (Table 5). Characteristics associated with decreased prevalence of IOL included: previous caesarean section, malpresentation and pre-term birth. IOL was less common for primiparous than multiparous mothers if they were non-Aboriginal, but more common if they were Aboriginal. The prevalence of IOL increased with increasing age for Aboriginal mothers but for non-Aboriginal mothers was slightly lower in older than for younger age-groups. Importantly, the evidence for the trend of increasing inductions over time among Aboriginal mothers remained after adjustments for other variables (Table 5).

**Table 5:** Adjusted odds ratios from mulitvariate logistic regression model of factors predictive of induction of labour among Northern Territory women who birthed in 2001–2012 by Aboriginal status and induction of labour status

	Aboriginal	Non-Aboriginal
Characteristic	OR (95 % CI)	OR (95 % CI)
Year	1.04 (1.02, 1.05)	1.00 (0.99, 1.01)
Maternal age		
Less than 20 years	0.76 (0.73, 0.96)	1.00 (0.85, 1.19)
20 to 24 years	0.84 (0.73, 0.96)	1.05 (0.96, 1.16)
25 to 29 years	1.00	1.00
30 to 34 years	1.12 (0.95, 1.31)	0.92 (0.85, 1.00)
35 years and over	1.05 (0.86, 1.28)	0.90 (0.85, 1.01)
Marriage status		
Single	0.78 (0.71, 0.86)	1.08 (0.99, 1.18)
Married/defacto	1.00	1.00
Other	1.07 (0.86, 1.33)	0.97 (0.88, 1.07)
Parity		
First-time mother	1.19 (1.06, 1.33)	0.85 (0.80, 0.92)
1–2 previous births	1.00	1.00
≥ 3 previous births	1.23 (1.09, 1.39)	1.37 (1.22, 1.54)
Place of birth		
Public hospital	1.00	1.00
Private hospital	3.02 (1.94, 4.67)	3.12 (2.90, 3.35)
Other	NA^a^	0.01 (0.00, 0.06)
Residence of mother		
Urban	1.00	1.00
Remote	0.98 (0.89, 1.09)	1.02 (0.92, 1.12)
Medical and obstetric factors^b^		
Multiple pregnancy	1.31 (0.76, 2.23)	1.36 (0.98, 1.87)
Early antenatal visit	1.11 (1.01, 1.22)	1.15 (1.07, 1.24)
Previous caesarean section	0.26 (0.22, 0.30)	0.14 (0.12, 0.16)
Smoked during pregnancy	0.81 (0.74, 0.89)	1.11 (1.02, 1.22)
Diabetes in pregnancy	3.49 (3.04, 4.02)	2.15 (1.90, 2.45)
Maternal hypertension	4.27 (3.50, 5.21)	6.45 (5.32, 7.83)
Pre-eclampsia	10.69 (9.01, 12.68)	9.78 (8.39, 11.41)
Antepartum haemorrhage	1.34 (0.95, 1.91)	1.28 (1.03, 1.59)
Malpresentation	0.17 (0.11, 0.25)	0.19 (0.15, 0.25)
Preterm delivery	0.46 (0.38, 0.56)	0.32 (0.26, 0.39)
Late term (≥41 weeks)	7.35 (6.48, 8.34)	6.16 (5.66, 6.71)
Premature rupture of membranes	2.21 (1.68, 2.90)	2.65 (1.99, 3.54)
Prolonged rupture of membranes	10.83 (9.15, 12.82)	13.11 (11.20, 15.35)
Intrauterine growth retardation	1.22 (1.08, 1.38)	1.11 (0.99, 1.24)
Macrosomia	1.19 (1.01, 1.40)	1.26 (1.15, 1.39)

^a^Odds ratio not applicable as there were no cases among induced mothers

^b^Medical and obstetric factors compared mothers with the factor to those without the factor

## Discussion

There are major differences between Aboriginal and non-Aboriginal mothers in the prevalence of, and factors associated with, induction of labour, and the changes in both over recent years. The prevalence of IOL increased for Aboriginal mothers, driven largely by increasing prevalence among primiparous mothers, which increased from 20.4 % to 30.3 %. Prevalence of IOL did not increase for non-Aboriginal women overall, although there were small changes in opposite directions for primiparous and multiparous mothers. In the last period of the study (2010–2012), the prevalence of IOL for Aboriginal women was only slightly lower than non-Aboriginal women, despite substantial differences in demographic characteristics, obstetric risk factors and co-morbidities between the groups. Furthermore, the increasing rate among Aboriginal mothers over this period could not be fully explained by the medical and obstetric indications for IOL analysed in this study.

Nationally, IOL rates changed little between 2001 (26.6 %) and 2011 (26.0 %), with proportions ranging from 22.6 % in the Australian Capital Territory, to 33.2 % in Tasmania [10]. In contrast, longitudinal data from Australia’s most populous state, New South Wales (where Aboriginal mothers account for less than 3 % of births) between 2001 and 2009 found an increase in IOL at all gestational ages, together with a corresponding decrease in definitive indications (e.g. hypertension and fetal distress) [15]. IOL rates have also increased markedly in Tasmania [9]. These states’ trends are in contrast to the stable prevalence of IOL among non-Aboriginal NT mothers.

Data about IOL for Aboriginal mothers elsewhere in Australia is limited. In Western Australia, 23.2 % of Aboriginal mothers and 28.9 % non-Aboriginal mothers underwent an IOL in 2011 [22]. In Victoria, rates were 22.7 % and 24.7 % respectively in 2011 [24].

For non-Aboriginal NT mothers, the decreased prevalence of key IOL drivers such as pre-eclampsia and hypertension and the rise in caesarean sections between 2001 and 2012 appear to have negated the increased prevalence of other key IOL drivers such as late-term pregnancies, prolonged rupture of membranes and diabetes.

For Aboriginal mothers, especially primiparous mothers, the answer to what drove the increasing IOL prevalence is not clear from these results. The prevalence of IOL among Aboriginal and non-Aboriginal women converged while risk factors for IOL did not. After adjustment, most factors retained the same direction of association to IOL for Aboriginal and non-Aboriginal mothers, but the strength of the association differed, some stronger in Aboriginal mothers (e.g. diabetes, IUGR), others in non-Aboriginal mothers (e.g. private hospital, previous caesarean section, hypertension, macrosomia). Smoking and primiparity were the only factors that had significantly opposite effects on the odds of IOLs between the two cohorts.

This study revealed alarming trends regarding smoking in pregnancy. Smoking among non-Aboriginal mothers halved between the first and last periods (20.6 to 11.1 %), but increased for Aboriginal mothers (from 40.5 to 46.5 %). As a modifiable risk factor in pregnancy outcomes, smoking cessation in pregnancy remains a very important issue for NT Aboriginal maternity services.

Post-dates as recorded in the MC (a recognised indication for IOL) remained the largest single cause of induction in the NT for both Aboriginal and non-Aboriginal mothers. However, the proportion of IOLs attributed to post-dates among non-Aboriginal mothers only increased marginally between 2001 and 2012, and did not change significantly among Aboriginal mothers. Prolonged rupture of membranes retained the strongest odds after adjustment (Table 5), but became less common as an obstetric complication (Table 3) and as a cause of IOL among Aboriginal mothers (Table 4) over the study period. The most dramatic change among clinical reasons for IOL was the increase in diabetes, though as the fifth and sixth most common reason for Aboriginal and non-Aboriginal mothers’ IOLs respectively, diabetes was a less important driver of IOL than other factors.

The convergence of IOL rates between Aboriginal and non-Aboriginal mothers may also be influenced by improved access and utilisation of health services by Aboriginal women in the NT, resulting in better diagnosis and intervention of medical and obstetric complications among these mothers. Aboriginal Community Controlled Health Organisations and NT Government Community Health Centres in remote Aboriginal communities have played an increasing role in providing antenatal care [25–28]. The Australian government has also made significant contributions towards pregnancy and early childhood programs [29]. But with the significant variability in the quality of antenatal care accessed by Aboriginal women in the NT [30, 31] it is difficult to assess the impacts of these services on obstetric care and management for Aboriginal mothers. Studies evaluating maternal and birth outcomes for Aboriginal mothers in the NT will form an important knowledge source to inform obstetric practice and maternity service policy.

Changing prevalence of IOL has important implications for pregnancy outcomes. In the presence of recognised risk factors, IOL can reduce the need for caesarean delivery and the risk of poor fetal outcomes [32, 33]. However, outside of these circumstances IOL has been linked to increased risk of emergency caesarean, vacuum extraction and need for epidural analgesia [5, 34]. A study from NSW found that the “IOL failure rate” (i.e. the need for caesarean or instrumental delivery after IOL) was six times higher among primiparous mothers [8]. A study on caesarean section trends in the NT between 1986 and 2012 demonstrated that IOL increased the likelihood of a caesarean section delivery with labour (comparable to emergency caesarean) for both Aboriginal and non-Aboriginal mothers, with a larger effect among primiparous mothers [35]. The increasing frequency of IOL among primiparous women in the NT, with a consequent increase in frequency of emergency caesareans, will impact on the frequency of elective caesareans in the future. These trends are already evident in the NT where the most common reason for elective caesarean section births is previous caesarean section (69 %)[21, 35].

A strength of this study is the reliable recording of Aboriginal status in the NT health records, which permits accurate comparisons between Aboriginal and non-Aboriginal Territorians [36]. A further strength is the use of the MC, a long-standing database that is maintained and validated by a dedicated Perinatal Business Analyst [21]. This validation process does include routine checking against medical records and patient discharge summaries from their birthing hospital, however many variables do not undergo this level of verification [21]. An example of this is the very high proportion of non-Aboriginal women whose marital status was recorded as ‘other’ between 2010 and 2012, which may represent an error in data management. A limitation of this study was that the characteristics analysed were restricted to those collected in the MC. One example of an important missing variable in this study is obesity and overweight, which is known to influence IOL prevalence [7]. This study excluded NT residents who birthed interstate. Mothers may birth interstate due to personal choice or medical necessity, but as interstate births are estimated to involved less than 2 % of NT mothers [21] their exclusion is unlikely to have introduced significant bias. This study included a substantial proportion of Aboriginal mothers, but we did not undertake direct engagement with the NT’s Aboriginal maternal community. It is our hope that this work prompts wider discussion among maternity services, researchers and Aboriginal communities.

A further limiting factor in this study was that the main indication for IOL was recorded as ‘other’ for one in four induced non-Aboriginal mothers and nearly one in five Aboriginal mothers throughout the study period. This figure is consistent with other Australian obstetric trend data [15, 37]. While the ‘other’ umbrella may include well-established clinical indicators for induction not individually listed on the MC (e.g. chorioamnionitis, isoimmunisation) [37], it is much more common than the expected prevalence of these relatively rare conditions. Instead, these ‘other’ reasons may represent decisions that were not based on prevailing clinical guidelines [13] or may also reflect the complexities of obstetric decision making, where factors like maternal request, practitioner ability, staffing, capacity pressures [38] and geographical remoteness must be taken into account. Review of clinical records might have provided more information for cases with undefined reasons for IOL.

## Conclusion

IOL has become more common for Aboriginal mothers birthing in the NT over recent years. By 2012 it was almost as common as for non-Aboriginal mothers, even though their demographic, medical and obstetric profiles were markedly different, suggesting that there are different influences affecting IOL related decisions for Aboriginal and non-Aboriginal mothers.

It would appear that the increasing frequency of IOL for Aboriginal mothers is being driven by increasing clinical complexity, but as the increasing trend among Aboriginal mothers in the NT could be not explained by definitive medical indications, it is imperative to ensure that the intervention was and continues to be associated with improved pregnancy outcomes rather than leading to unintended consequences, most importantly emergency caesarean section.

## Abbreviations

CI: confidence interval
IOL: induction of labour
IUGR: intrauterine growth restriction
MC: Midwives collection
NT: northern territory
ROM: rupture of membranes
USA: United States of America
